# Pathogenicity island *cag*, *vacA *and IS*605 *genotypes in Mexican strains of *Helicobacter pylori *associated with peptic ulcers

**DOI:** 10.1186/1476-0711-10-18

**Published:** 2011-05-13

**Authors:** Fernando Antonio-Rincón, Yolanda López-Vidal, Gonzalo Castillo-Rojas, Eduardo C Lazcano-Ponce, Sergio Ponce-de-León, María L Tabche-Barrera, Germán R Aguilar-Gutiérrez

**Affiliations:** 1 Centro de Investigaciones Sobre Enfermedades Infecciosas, Instituto Nacional de Salud Pública. Cuernavaca, Morelos, México; 2 Programa de Inmunología Molecular Microbiana, Departamento de Microbiología y Parasitología, Facultad de Medicina, UNAM, México, DF, México; 3 Centro de Investigaciones en Salud Poblacional, Instituto Nacional de Salud Pública, Cuernavaca, Morelos, México; 4 Instituto de Biotecnología, UNAM, Morelos, Cuernavaca, Morelos, México; 5 Unidad de Epidemiología Clínica, Instituto Nacional de Ciencias Médicas y Nutrición Salvador Zubirán (INCMNSZ), México, DF, México

**Keywords:** *Helicobacter pylori*, *cag *PAI, *vacA*, peptic ulcers, Mexico

## Abstract

**Background:**

*Helicobacter pylori *is associated with chronic gastritis, peptic ulcers, and gastric cancer. Two major virulence factors of *H. pylori *have been described: the pathogenicity island *cag *(*cag *PAI) and the vacuolating cytotoxin gene (*vacA*). Virtually all strains have a copy of *vacA*, but its genotype varies. The *cag *PAI is a region of 32 genes in which the insertion of IS*605 *elements in its middle region has been associated with partial or total deletions of it that have generated strains with varying virulence. Accordingly, the aim of this work was to determine the *cag *PAI integrity*, vacA *genotype and IS*605 *status in groups of isolates from Mexican patients with non-peptic ulcers (NPU), non-bleeding peptic ulcers (NBPU), and bleeding peptic ulcers (BPU).

**Methods:**

The *cag *PAI integrity was performed by detection of eleven targeted genes along this locus using dot blot hybridization and PCR assays. The *vacA *allelic, *cag *PAI genotype 1 and IS*605 *status were determined by PCR analysis.

**Results:**

Groups of 16-17 isolates (n = 50) from two patients with NPU, NBPU, and BPU, respectively, were studied. 90% (45/50) of the isolates harbored a complete *cag *PAI. Three BPU isolates lacked the *cag *PAI, and two of the NBPU had an incomplete *cag *PAI: the first isolate was negative for three of its genes, including deletion of the *cagA *gene, whereas the second did not have the *cagM *gene. Most of the strains (76%) had the *vacA *s1b/m1 genotype; meanwhile the IS*605 *was not present within the *cag *PAI of any strain but was detected elsewhere in the genome of 8% (4/50).

**Conclusion:**

The patients had highly virulent strains since the most of them possessed a complete *cag *PAI and had a *vacA *s1b/m1 genotype. All the isolates presented the *cag *PAI without any IS*605 *insertion (genotype 1). Combined *vacA *genotypes showed that 1 NPU, 2 NBPU, and 1 BPU patients (66.6%) had a mixed infection; coexistence of *H. pylori *strains with different *cag *PAI status was observed in 1 NBPU and 2 BPU (50%) of the patients, but only two of these patients (NBPU and BPU) had different *vacA *genotypes.

## Introduction

*H. pylori *is a well-recognized pathogen that chronically infects the stomach of up to 50% of the world's human population. The prevalence of *H. pylori *is high in developing countries; in Mexico its seroprevalence is 66% of the general population and is common in asymptomatic population [1-5].

There are two genotypic characteristics of virulent *H. pylori *strains: the *vacA *gene, and the *cag *PAI region. Virtually all *H. pylori *strains have a copy of *vacA*, but the structure among alleles varies in three regions: the signal (s) region that is present as type s1 (subtype a, b and c) or type s2, the intermediate (i) region that exists in subtype 1 and 2, and the middle (m) region that exists in three different allelic forms (m1, m2a, and m2b). The final structure of the *vacA *gene is an extensive allelic diversity where all combinations of all those regions have been reported. The s1/m1 genotype encodes a VacA protein, which possesses high vacuolating cytotoxin activity and is associated with severe gastroduodenal diseases [6-9]. Infection by multiple *H. pylori *strains, defined by different *vacA *genotypes, is common in Mexico. Nevertheless, the most prevalent *vacA *genotypes in the Mexican population are s1(b)/m1 and s2/m2, which have been associated with the development of chronic gastritis (CG), peptic ulcers (PU), and gastric cancer (GC) [10-17].

*cag *PAI encodes components of a type IV secretion system (T4SS), which is used for the translocation of CagA into the gastric epithelium cells, where it is phosphorylated. Phosphorylated CagA interferes with various physiological transduction signals in the host cell and causes pathological cellular responses that contribute to the development of local inflammation and the damage of the gastric mucosa. The elimination of T4SS function significantly reduces the accumulation of phosphorylated CagA in the host cell and the secretion of interleukin 8 (IL-8) [18]. The presence of *cag *PAI has been associated with the development of CG, PU, and GC [19].

In Mexico, studies of *cag *PAI and its relationship with the infection outcomes have focused on the *cagA *gene and its product, protein CagA, which have been used as biomarkers of this genetic region. These studies have shown a correlation between serologic detection of antibodies against CagA and the identification of the infecting *H. pylori cagA*+ strains [2-4,12,20,21]. A series of studies have shown a close association between CagA antibodies and the development of gastroduodenal pathologies such as PU and GC [12,22,23]. The presence of *cagA *as a biomarker for *cag *PAI and gastric colonization with *cagA*+ strains associated with gastrointestinal diseases has been documented by PCR in isolated bacterial DNA and has also been detected by hybridization *in situ *from biopsies or by microarray hybridization [4,5,10-12,14-16,24,25].

Nevertheless, many studies have demonstrated that T4SS integrity is needed for translocation of CagA protein into gastric epithelium cells and that the insertion of IS*605 *elements between cag I and cag II of *cag *PAI has been implicated in partial or total deletions that have generated *H. pylori *strains with varying virulence [26,27].

In Mexico three studies have addressed the correlation between genetic integrity in *cag *PAI in multiple isolates obtained from individual patients with gastroduodenal diseases. One study characterized the *vacA *genotypes and evaluated the presence of *cag *PAI by PCR amplification of the *cagA *in four children with chronic abdominal pain (CAP), eight adult patients with PU and one with non-ulcer dyspepsia (NUD). This study also characterized the diversity of *cag *PAI by microarray genotyping in 51 isolates from three of the adult with PU and four of the pediatric patients [16]. The second study showed homogeneous *cag *PAI+ content in eight adults and ten pediatric patients with CAP, PU or NUD, after evaluating the presence of *cagA*, *cagE*, *cagT *and *cag10 *genes by PCR [25]. The third study, which used DNA microarrays, showed that the presence of a complete *cag *PAI was significantly more frequent for isolates from patients with GC than for isolates from non-atrophic gastritis (NAG) and duodenal ulcer (DU) patients [5]. Nevertheless, the integrity of *cag *PAI and *vacA *genotypes as important contributors to the risk of PU development, and its most common complication, BPU, had not previously been studied in the Mexican population. Accordingly, the goal of this work was to establish the *cag *PAI*, vacA and *IS*605 *status in groups of single-colony isolates from Mexican patients with non-peptic ulcers and (bleeding or not bleeding) peptic ulcers.

## Methods

### Study subjects

Two patients with gastric or duodenal ulcers with ongoing or recent (24 h) bleeding (BPU) were recruited and the gastric biopsy samples from each were taken into the first 24 hours. Four additional patients were included in the study as controls: two patients with NBPU and two on whom an endoscopy was performed because of non-ulcer dyspepsia and/or gastro-esophageal reflux (NPU). None of the patients had clinical history of mucosa associated lymphoid tissue (MALT) lymphoma or Crohn's disease. 50 clinical *H. pylori *isolates were obtained from biopsy specimens: 17 (8 and 9) from the two patients with BPU; 17 (12 and 5) from the two patients with NBPU, and 16 (9 and 7) from the patients with NPU. All six patients were recruited from a Mestizo-Mexican population in a previous cross-sectional, prospective, observer-blind study carried out between November 1995 and August 1997 [4]. The subjects ranged from 53 to 72 years old (mean 61 years), and the F/M sex ratio was 1:1 in the NBPU and NPU groups and 0:2 in the BPU group. At least seven biopsies from the antral and corpus gastric mucosa were taken during the same endoscopic sessions of each patient.

### *H. pylori *culture and chromosomal DNA extraction

*H. pylori *was cultured by smearing biopsy specimens on the surfaces of horse blood agar plates. The colonies obtained were identified as *H. pylori *according to standard criteria, including typical cell morphology, negative Gram staining, and biochemical testing. The rest of the colonies from each plate were harvested in batches to obtain a new culture on blood agar plates. Between 9 and 12 colonies were picked separately from these plates and were then passed onto individual plates. Each of these plates was propagated three more times for the obtaining of biomasses and extraction of chromosomal DNAs as previously reported [10]. In the current study these chromosomal DNAs were used to carry out all the genotyping assays after their characterization by PCR amplification of the *H. pylori *16S rRNA gene, using the HP16-219 and HPGR-16S pair of primers (see Table 1).

**Table 1:** Primers used in this study for *H. pylori *genotyping.

Primer	Sequence (5'-3')	Size of amplified fragment	Annealing temperature °C	Position in genome of reference (*H. pylori *26695)*
**16S rRNA**				
HPGF-16S	CAATCAGCGTCAGTAATGTTC	516	55	1208359-1208379
HPGR-16S	CTAAGAGATCAGCCTATGTCC			1208855-1208875
***cag *PAI genes**				
cag1-F	TTCATTAGGTCTCATTGGAGCAGG	230	62	547429-547452
cag1-R	TTGGCTTCAGTTGGTTCGTTG			547639-547659
cag1-F	TTCATTAGGTCTCATTGGAGCAGG	430	66	547429-547452
cag1-2-R	CCCTTACAGCCGCCTTTATTTAC			547837-547859
cag5-F	CGCCACTCTTATCTTCTTCACCTC	645	66	551152-551175
cag5-R	GGGGATTTTATCGCTTACGCAG			551776-551797
cag7-F	AAGCGTGTCATAATGGGTGTGC	986	66	554726-554747
cag7-R	AGAAAAGGCTGTTGCGGATTG			555692-555712
cag9-F	ATGTCCCCCCAACAAATCGC	627	62	562353-562372
cag9-R	CCATTAGCCACAAGTTTAGCCG			562959-562980
cagT-F	GTTCACCATTCTAAAAAGATTACGC	555	66	565331-565355
cagT-R	GATTTCGCAAGTATTCATTCTCTC			565863-565886
cagS-F	CAATGGTTTTTAGATTAGCG	342	54	566203-566222
cagS-R	AAGGGAGCGTTAGATAAGG			566527-566545
cagS-Q-F	TCTAACGCTCCCTTGTTTGTATGC	687	66	566532-566555
cagS-Q-R	TTGGTTGGTAATGGTTTTGGTAGC			567196-567219
cagQ-F	CCAAAACCATTACCAACCAAAGC	292	66	567200-567222
cagQ-R	CCGAACAAGCAAGAACTTACACAAC			567468-567492
cagM-F	GGTTGCGTTTGGAGTTTTGTCG	485	66	568752-568773
cagM-R	TGAGCCTTATCTTTAGTGGTAGCGG			569213-569237
cagI-F	TGTTCTTCCCAAAGGTCGGC	510	62	571644-571663
cagI-R	CTTCTGACAACGCTCAATACATCG			572131-572154
cagE-F	AGTGATGCTTTGAGTCGCAAGTC	923	64	575506-575528
cagE-R	TGGGGCAATAGTGTGATGACG			576409-576429
cagA-F	GCCTAATTTAAATAATCTCGCTATCAC	744	55	581555-581581
cagA-R	ATTGAGATTGTCAACTTTATCCG			582277-582299
IS605-F	TTTGATAAAAACGGATGTGTGG	1,016	55	13986-14007 (AC000108)#
IS605-R	TGTTTTTGCAATAAGGGGAT			14983-15002
TnpB-cagS-F	CGTTCTTAAGCCCATGTCTAAACC	1,597	56	14038-14061 (AC000108)#
TnpB-cagS-R	TCTAACGCTCCCTTGTTTGTATGC			15612-15635

*[GenBank: AE000511.1]; #[GenBank: NCTC11638].

### PCR Primer Design

Using the genome sequence of the *H. pylori 26695 *strain as reference (ATCC 700392) [GenBank: AE000511.1] [28], pairs of primers were designed to carry out specific PCR amplifications for one or two of selected genes from the region cag I: *cag6, cag6-7, cag10, cag13 *and *cag16*, *cagT *and *cagS; *and from the cag II region of *cag *PAI: *cagQ, cagM, cagI, cagE *and *cagA *[26,27]. The cagS-Q-F and cagS-Q-R pair of primers was designed to amplify the DNA cagS-Q fragment and to detect the presence of the IS*605 *between the *cag *I and *cag *II regions of *cag *PAI. The TnpB-cagSF and TnpB-cagSR primers were designed to detect the IS*605 *contiguous to the *cag *II region that characterizes the genotype 2 of *cag *PAI previously reported [26]. To determine if IS*605 *was present elsewhere in the genome, the primers IS605F and IS605R were used. For amplification of *cagA*, the primers cagA-F and cagA-R were designed from a consensus sequence of a set of 26 *cagA *gene-sequences reported [GenBank: AB015404-16; AF202972-3; AB011791-2; AF249275; AF001357; L11714; AF083352; AF282853; AF247651; AE001483 and AB003397], using the MacVector 2.1 molecular biology program. Table 1 shows the nucleotide sequence of PCR primers used for the generation of all these DNA-fragments. The parameters for the amplification of IS*605 *in the genome were 94°C for 45 seconds, 54°C for 1 minute and 30 seconds, and 72° C for 1 minute and 30 seconds, for a total of 30 cycles. The amplification parameters for DNA fragment *cagS*-Q were 94°C for 1 minute, 50°C for 1 minute, 72°C for 2 minutes, for a total of 30 cycles.

### Cloning of the *cag *PAI genes

Eleven DNA fragments corresponding to individual selected *cag *PAI genes and two DNA fragments that included the *cag6-7 *and *cagS*-*cagQ *genes were generated by PCR. The thermal cycling conditions were at 94°C for 30 seconds, the primer annealing temperature ranging from 54-66°C (depending on the melting temperature of each set of primers that were used; see Table 1) for 30 seconds, and 72°C for 30 seconds, for a total of 30 cycles. The PCR products were cloned directly into the linearized pCR^®^2.1-TOPO^® ^vector to obtain a genetic library of *cag *PAI formed by thirteen plasmids. The thirteen cloned fragments showed 100% identity in their nucleotide sequence when they were compared with the reported sequence of the *H. pylori *strain *26695*, using the BLAST program.

### Southern blot hybridization

For all the *H. pylori *strains and controls, 500 μg of genomic DNA were transferred to a nylon membrane (Hybond-N+) with sodium hydroxide. The genomic DNAs were hybridized with each one of the plasmids of the genetic library of *cag *PAI, which were previously digested with the *Eco*RI overnight at 37°C. The plasmid DNAs were labelled with thermostable alkaline phosphatase, Alkphos Direct Labelling and Detection System and CDP-Star (Amersham Pharmacia Biotech). The membrane was pre-hybridized for 2 hours at 55°C, and hybridized at 55°C overnight. The membrane was washed three times at 58°C, in a shaking water bath for 15 minutes, transferred to a clean container and washed twice with secondary wash solution for 10 minutes at room temperature. Excess of wash solution was removed from the membrane and the CDP-Star substrate was added and incubated at room temperature for 5 minutes. Afterwards the membrane was wrapped in plastic film and autoradiography was performed with a sheet of Hyperfilm™ ECL (Amersham Pharmacia Biotech) at different lengths of time. The membranes were stripped from the previously hybridized probe and stored in hybridization bags at 4°C to be used with another probe.

### *vacA *genotyping by specific PCR amplification

The *vacA *signal and middle regions were typed by using the primers as described previously by Atherton *et al*. [6]. The isolates were at first identified as type s1 or s2 and type m1 or m2. All strains with signal region type s1 were further characterized into s1a or s1b variants by performing three separate PCR assays. *H. pylori 26695 *(*cag *PAI+, *vacA *s1a/m1), J99 (ATCC 700824) (*cag *PAI+, *vacA *s1b/m1), Tx30a (ATCC 51932) (*cag *PAI-, *vacA *s2/m2), and 86-313 (*cag *PAI-, *vacA *s2/m2) strains were used as controls for *vacA *allele detection [28-30]. Distilled water was used as an internal-reaction negative control. Thermal cycling conditions for each set of primers (0.5 μM) were at 95 °C for 1 minute, 52 °C for 1 minute, for a total of 35 cycles.

## Results

**Figure 1:**
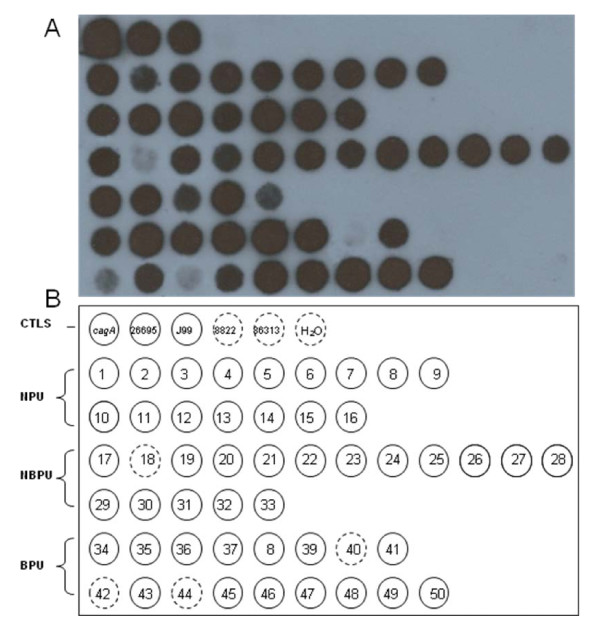
**Detection of the *cagA *gene by Southern blot hybridization**. (A) Hybridization for 50 genomic DNA of the isolates and 4 *H. pylori *reference strains as positive and negative controls, as well as a control the plasmid with the intra-*cagA *gene cloned, and distilled water as an internal-reaction negative control. (B) Schematic representation of the result of hybridization: circles continuous and discontinuous for positive and negative hybridization, respectively. Abbreviations: CTLS, controls; NPU, non-peptic ulcers; NBPU, non-bleeding peptic ulcers, and BPU, bleeding peptic ulcers.

We investigated the integrity of *cag *PAI, *vacA *alleles, and the presence of IS*605 *in groups of single-colony isolates from three pairs of Mexican patients with NPU, NBPU, and BPU. To analyze the integrity of the *cag *PAI region of groups of 5-12 single-colonies (n = 50), Southern blot hybridizations were performed. The analysis was based on the detection of eleven targeted genes homogeneously distributed along this locus using the *cag *PAI genetic library as a probe. An example of detection of the *cagA *gene by hybridization is illustrated in Figure 1. To confirm the result of the hybridizations, the genomic DNAs were used for detection of *cagA *gene by PCR using the pair of consensus primers described in Table 1. The percentage of detection of *cagA *gene by PCR, with respect to those identified by hybridization, was 97.7%. Thus, a gene marker was defined as positive if it was detected by dot blot and not by PCR. The results showed that 90% (45/50) of the isolates were positive for all of the studied genes of *cag *PAI, whereas 4% (2/50) and 6% (3/50) had an incomplete *cag *PAI or were negative for all the analyzed genes, respectively. Co-existence of strains with different *cag *PAI status was observed in 50% (3/6) of the patients (see Table 2). 2 BPU patients were infected with multiple strains, some of which had a complete *cag *PAI, and others that didn't: 1/8 (12.5%) and 2/9 (25%), respectively. Meanwhile, 1 NBPU patient was infected with strains with a complete *cag *PAI but also with strains with an incomplete island 2/12 (16%). One of these strains showed deletions of the *cagW, cagT, cagI*, including deletion of the *cagA *gene, while the other did not have the *cagM *gene. Thus, the presence of the complete *cag *PAI was found in 100% (16/16); 88.2% (15/17); 82.3% (14/17) of the clinical isolates from the group of patients with NPU, NBPU, and BPU, respectively. The results of the Southern hybridization were confirmed by PCR amplifications of the eleven *cag *PAI gene markers in the five strains that had the incomplete or lacked the cag PAI. The PCR amplifications were similar to those obtained by the hybridization method.

**Table 2:** Association between integrity *cag *PAI, IS*605*, and *vacA *genotype of *H. pylori*.

***vacA *genotype**								
		
***cag*PAI**	**IS*605 *present outside *cag *PAI**	**Non-peptic ulcers**		**Non-Bleeding Peptic ulcers**		**Bleeding peptic ulcers**		**Total % of isolates**
		**Patient 1**	**Patient 2**	**Patient 1**	**Patient 2**	**Patient 1**	**Patient 2**	
		
CP +	-	s1b/m1 (9)*	**s1b/m2 (5), s0/m1 (1)**	**s1b/m1 (7) s2/m2 (1)**,	**s1b/m1 (3), s1b/m2 (1), s0/m1 (1)**	**s1b/m1 (7)**	**s1b/m1 (6), s1b/m0 (1)**	84
CP +	+		**s1b/m2 (1)**	**s1b/m1 (2)**				6
CP -	-					**s1b/m1 (1)**	**s1b/m1 (2)**	6
CP +/-	+			**s0/m1 (1)**				2
CP +/-	-			**s1b/m1 (1)**				2

( )*, numbers of isolates; CP+, complete; CP-, without; CP+/-, incomplete; s0, untypeable for signal region; m0, untypeable for middle region. Patient No.1: Mestizo female, 54 years old, without peptic ulcers or ulcer history; patient No 2: Mestizo male, 53 years old, without peptic ulcers or ulcer history; patient No 3: Mestizo male, 63 years old, with ulcer history, non-bleeding duodenal ulcers; patient No 4: Mestizo female, 65 years old, with ulcer history, non-bleeding duodenal ulcers; patient No 5: Mestizo male, 61 years old, without ulcer history, bleeding duodenal ulcers; patient No 6: Mestizo male, 72 years old, with ulcer history, bleeding gastric ulcers.

PCR analysis was performed in order to determine whether the clinical isolates had a pathogenicity island that was uninterrupted or one that was divided in two regions as the result of insertion of a single-copy of IS*605 *element, similar to genotypes 3 described previously [26]. These PCR assays were carried out using specific primers for sites upstream and downstream of this insertion (cagS-Q-F and cagS-Q-R; Table 1) to amplify the *cagS*-*Q *DNA fragment. DNA fragments of 2,567 pb or 687 pb were predicted whether or not the *cag *PAI was split by an IS*605 *element. The results of this analysis showed the presence of a *cag*S-Q fragment of 687 pb in 100% (16/16) of the isolates of the patients with NPU. In the group of patients with BPU, 70.5% (12/17) of the isolates resulted positive for this DNA fragment. Three fifths (3/5) of isolates that were negative for this DNA fragment were also negative for the *cag *PAI by dot blot hybridization (11.7%) (*cag *PAI- isolates; see Table 2).The 2/5 remaining BPU isolates were negative for the *cag*S-Q DNA fragment, even though they were positive for *cag*S and *cag*Q genes by dot blot hybridization. Similarly, one isolate from a NBPU patient (1/9) was negative for this fragment but positive for *cagS *and *cagQ *genes by Southern blot. PCR amplifications were performed in order to rule out the possibility that these three strains may have had an IS*605*-based rearrangement in the island similar to genotype 2 in the NCTC 11608 strain (where the *cag *I and *cag *II regions are separated by a large fragment of chromosomal DNA flanked by two IS*605 *sequences) [26,27]. The *tnp*B-*cag*S-F and *tnp*B-*cag*S-R primers were used to amplify a 1,597 pb DNA fragment between the *cagS *gene and the *tnpB *transposases of the adjacent IS*605*, using the NCTC 11608 strain as a positive control. The IS*605 *insertion sequence was not found, and it can therefore be assumed that the *cag *PAI was presented as an uninterrupted unit in 94% (47/50) of the analyzed isolates (*cag *PAI 1 genotype) [26].

In this study, 2 strains (4%) presented partial deletions of *cag *PAI. The presence of IS*605 *anywhere in the *H. pylori *chromosome is thought to be involved in *cag *deletions [26].To determine whether or not the clinical isolates had the IS*605 *insertion elsewhere in the chromosome, the IS605-F and IS605-R primers were used to amplify a *tnp*A-*tnp*B DNA fragment of 1,016 pb. The results of this analysis showed that the IS*605 *insertion was present in 8% (4/50) of the isolates. One isolate with a complete *cag *PAI was present in a patient with NPU, and three isolates were present in only one patient with NBPU: two of the isolates had a complete *cag *PAI and another had an incomplete *cag *PAI. Thus, the complete-*cag *PAI/IS*605*- genotype of the strains was predominant in the different patients in this study.

To account for the possibility that the different patients might have been infected with multiple strains of *H. pylori*, we investigated the *vacA *genotypes of the clinical isolates. Three combinations of *vacA *s and m regions were identified: 38 (76%) of the strains had the s1b/m1 genotype that was previously described as the most prevalent *vacA *genotype in Mexican populations [10,14]. The second most frequent *vacA *genotype was s1b/m2, which was present in 7 (14%) strains, whereas the s2/m2 genotype, previously reported as the most prevalent genotype in PU patients from north-eastern of Mexico [11,12], was only present in 1 (2%) of NBPU isolates. The s2/m1 genotype described previously was not identified [10-12,15]. Untypeable *vacA *genotypes have been previously described in Mexican populations. In this study we found 3 (6%) strains where *vacA *region m1 was amplified, but *vacA *s region was not detected (1 NPU, and 2 NBPU isolates). Likewise, 1 BPU isolate (2%) was s1b/m0, which accounts for the possibility that an additional subfamily of s and m genotypes might exist in Mexican isolates, given that in this study as in those reported elsewhere in México, the s1c and m3 *vacA *alleles, which have been described in some populations [31], were not investigated [10,11,14,17]. In this study, evidence of infection by more than one *H. pylori *strain was found in 66.6% (4/6) of the patients, where at least two *vacA *allotypes were detected. This confirms the multi-strain nature of the *H. pylori *infection previously described in the Mexican population [10,12-14] (see Table 2).

## Discussion

In this study which used eleven selected *cag *PAI genes as a probe to carry out the characterization of cag PAI by Southern blot hybridization, we found that among the 50 *H. pylori *isolates, a completed *cag *PAI was detected in 45 (90%) of them; 2 (4%) presented an incomplete *cag *PAI, whereas in 3 (6%) the *cag *PAI was not detected. Thus, most of our isolates possessed a complete *cag *PAI. Three previous Mexican studies are consistent with our results. The first of these, which evaluated the presence of four *cag *PAI genes markers by PCR assays, showed that 78.6% of the studied isolates had a complete *cag *PAI, while 15.1% did not have it, and 6.2% had partial deletions [25]. The second study, which used hybridization microarrays to characterize this genetic locus, showed that 18/29 (62%) of the studied isolates had a complete *cag *PAI [5]. Nevertheless, these two studies and those previously published about the Mexican population did not carry out the correlation between the integrity of *cag *PAI and *vacA *genotypes. Our data showed that 90% of the strains had a complete *cag *PAI and that the major *vacA *s1b/m1 and s1b/m2 genotypes were present in 34/50 (68%) and 7/50 (14%) of the strains, respectively, whereas minor s2/m2, s1b/m0 and s0/m1 genotypes were present in 1 (2%), 1 (2%) and 2 (4%). In 2/50 (4%) of the isolates with an incomplete *cag *PAI, the s1b/m1 and s0/m1 genotypes were present. Meanwhile, the *vacA *genotype was s1b/m1 in the 3/50 (6%) of the strains with an undetected *cag *PAI. Thus, strains with a complete *cag *PAI and *vacA *s1b/m1 genotype were the most common. In one of the NPU patients, the clinical isolates were highly homogenous: 100% (9/9) of the isolates had a complete island with a *vacA *s1b/m1 genotype (see Table 2). Nevertheless, 4/6 (66.6%) of the studied subjects had multiple genotype infections. The most notorious of them was one of the NBPU patients who not only had three strains with different *vacA *genotypes, including the most prevalent genotype in north-eastern of Mexico, the s2/m2, and untypeable s0/m1, but also 10/12 strains with a complete *cag *PAI, and 2/12 incomplete islands (see Table 2). One of these last strains showed deletions of the *cagW, cagT, cagI*, including deletion of the *cagA *gene, while the other strain, the *cagM *gene was deleted. In addition, this NBPU strain represented 1/4 of the strains which had an IS*605 *insertion somewhere in the chromosome. The deletions in our isolates are quite different from that reported previously [5], which showed that almost 24% of the deletion in *cag *PAI corresponded to the *cag2 *gene and has been reported as nonessential for functional activity of theT4SS [18].

Our data showed for first time that the 94% of the Mexican isolates had the *cag *PAI genotype 1 described previously [26], which did not have an IS*605 *insertion between cagI and cagII regions; the remaining 6% of the isolates lacked the *cag *PAI.

## Conclusion

The presence of the *cag *PAI and *vacA *genotypes in different subjects was analyzed according to disease group: no differences were found between BPU and NBPU isolates, in which the complete *cag *PAI was present in 88.2% and 82.3%, respectively. The presence of *vacA *s1b/m1 genotype in NBPU (76%) is different when compared with BPU (94%). Thus, we can conclude that although the integrity of *cag *PAI is similar in NBUP and BPU isolates, the presence of *vacA *s1b/m1 genotype in BPU has a slightly higher presence when compared with NBPU isolates.

## Competing interests

The authors declare that they have no competing interests.

## Authors' contributions

GCR, FAR, MLTB carried out the molecular genetic studies, and microbiological procedures. GCR, ECLP, SPDL analyzed and interpreted the data, and help to drafting the manuscript. GRAG, YLP conceived of the study, participated in its designed and coordination of the experiments, and drafted the manuscript. All authors read and approved the final manuscript.
